# Evaluation of serum uric acid level in systemic lupus erythematosus patients with normal and high pulmonary arterial hypertension

**DOI:** 10.1051/bmdcn/2018080316

**Published:** 2018-08-24

**Authors:** Miramir Aghdashi, Mahsa Behnemoon, Jila Mahmoodi Rad, Masoumeh Rabiepour

**Affiliations:** 1 Urmia University of Medical Sciences Urmia Iran

**Keywords:** Pulmonary arterial hypertension, Serum uric acid, Systemic lupus erythematosus

## Abstract

Background: Systemic lupus erythematosus (SLE) is a life-threatening multisystem inflammatory condition affected any organ system. Considering the role of uric acid as a pro-inflammatory compound in high pulmonary arterial pressure, serum levels of uric acid and its relation to severity and duration of the disease were assessed in SLE patients.

Methods: As a cross-sectional study, 75 patients with SLE were enrolled in Imam Khomeini Hospital and Sahand Clinic. Serum uric acid level was measured by pars azmoon kit. Pearson correlation coefficient and *T*-test were used for statistical analysis of data.

Results: The mean duration of SLE was 56.44 ± 40.57 months. High serum uric acid was observed in 13.3% of patients with SLE. Moreover, 8% of these patients had high pulmonary arterial pressure. Serum uric acid in patients with high pulmonary artery pressure was significantly higher than patients with normal pulmonary artery pressure *(P* < 0.01). Furthermore, a significant relation was seen between severity of SLE disease with serum uric acid level and pulmonary artery systolic pressure (*P* < 0.05). However, there was no significant correlation between serum uric acid level and duration of the disease (*p* = 0.90, r = 0.016).

Conclusion: According to these results, a significantly increased level of serum uric acid was observed in patients with pulmonary arterial pressure. Hence, serum uric acid level could be a prognostic marker of pulmonary arterial pressure in SLE patients which correlates with disease severity. It also would help to reduce clinical demands for echocardiography in patients with normal uric acid levels.

## 1. Introduction

Systemic lupus erythematosus (SLE) as a multisystem autoimmune disorder has diverse phenotypic expression affecting any organ system. Kidney, heart and pulmonary system are the most frequently affected organ in systemic lupus erythematosus [1]. Our understanding of the etiology of autoimmune diseases such as SLE remains unknown. Genetic, hormonal and environmental factors and immune deficiencies appear to be effective [2]. Studies reported that autoimmune diseases affect 7% of the population in the United States. Moreover, pulmonary arterial hypertension (PAH) [3, 4] is the third leading cause of mortality among patients with systemic lupus erythematosus. Other studies also reported that PAH was considered as the most frequent cause of mortality among these patients [5, 6]. The prevalence of pre pulmonary hypertension in SLE is about 1.8%-14% [4]. However, multiple factors are implicated in increased pulmonary hypertension in SLE disease, but the cause is vague [1]. Uric acid as the final product of purine oxidation is produced from xanthine by xanthine oxidoreductase (XOR) during the metabolism of components including DNA, RNA and ATP [5]. Serum uric acid can be detected through the balance between generation and reabsorption by the kidney and secretion by the intestine [6]. It shows excellent antioxidant activity at physiologic concentrations [7]; however, high level of uric acid may be due to local tissue ischemia and/or hypoxia or other factors [5]. Moreover, high level of uric acid is associated with increased oxidative disorder, severity of disease and inflammation in patients with high pulmonary hypertension [8]. Studies showed that uric acid triggers the release of several pro-inflammatory molecules such as monocyte chemoattractant Protein-1 and C-reactive protein in vascular cells. It is interesting that elevation of uric acid can play a main role in theprogression of PAH [9]. Some studies have shown that high level of uric acid is observed in patients with lupus and high pulmonary arterial pressure [10]. Considering the role of uric acid as pro-inflammatory compound in SLE disease and the relationship between high pulmonary arterial pressure and the severity of the disease [11] and lack of sufficient studies in our country, the aim of this study was to measure serum uric acid level in patients with systemic lupus erythematosus and its association with duration and severity of the disease.

## 2. Methods

### 2.1. Study design and population

In current study, we designed an analytical cross-sectional study and 75 patients with systemic lupus erythematosus were enrolled in Imam Khomeini Hospital and Sahand Clinic, Urmia, Iran during 2016-2017. Informed consent was taken from all participants. Moreover proposal was approved by the ethics committee of Urmia University of Medical Sciences.

### 2.2. Inclusion and exclusion criteria

In this study, inclusion criteria were having Systemic lupus erythematosus. Moreover, patients with previous history of hypertension, heart valve disease, thromboembolism, chronic obstructive pulmonary disease, gout, heart failure (EF < 40%) and kidney failure were excluded from the study.

### 2.3. Measurement of PAH and serum uric acid

After measuring pulmonary artery pressure in patients with echocardiogram, they were divided into two groups (high pulmonary arterial pressure and normal). Moreover, blood samples were taken from each patient. Then blood serum was separated by centrifuge and serum uric acid measured by Pars Azmoon Kit. Other data including severity of disease and duration of illness was extracted from patients’ records. Ultimately, the relationship between uric acid with pulmonary arterial pressure, severity and duration of the disease was evaluated.

### 2.4. Statistical Analysis

The data were entered into SPSS version 19 and Pearson correlation coefficient, *T*-test and Anova test were used for analysis of data.

## 3. Results

### 3.1. Demographic characteristics of the study population

In this study, among 75 patients with systemic lupus erythematosus 5 patients (6.7%) were male and 70 (93.3%) female. The mean duration of lupus was 56.44 ± 40.57 months.

### 3.2. Frequency distribution of patients in terms of UC, PAP and severity of disease

Table 1 shows frequency distribution of patients with systemic lupus erythematosus in terms of serum uric acid.

**Table 1:** Frequency distribution of patients with systemic lupus erythematosus in terms of serum uric acid.

Serum Uric acid level	Frequency	Percent
Normal serum uric acid	65	86.7
High serum uric acid	10	13.3
Total	75	100

As shown in Table 1, high serum uric acid was observed in 13.3% of patients.

Frequency distribution of patients with systemic lupus erythematosus in terms of pulmonary arterial pressure is shown in Table 2.

**Table 2:** Frequency distribution of patients with systemic lupus erythematosus in terms of pulmonary arterial pressure.

Pulmonary artery pressure	Frequency	Percent
Normal	69	92
High	6	8
Total	75	100

As shown in Table 2, 8% of patients high pulmonary arterial pressure.

Table 3 shows frequency distribution of patients with systemic lupus erythematosus in terms of severity of disease.

**Table 3:** Frequency distribution of patients with systemic lupus erythematosus in terms of severity of disease.

Severity of the disease	Frequency	Percent
Low severity of the disease	4	5.3
Moderate Severity of the disease	63	84
High severity of the disease	8	10.7
Total	75	100

As shown in Table 3, high severity of the disease was observed in 10.7% of patients.

Table 4 shows comparison between patients with normal and high pulmonary artery pressure in terms of serum uric acid.

**Table 4:** Comparison of patients with and without pulmonary artery pressure in terms of serum uric acid.

Variables	Serum uric acid	*P*-value
	Mean ± SD	`*T* - test
Normal pulmonary artery pressure	4.19 ± 1.74	
		0.001
High pulmonary artery pressure	6.20 ± 2.13	

As shown in table 4, significant difference was observed between patients with normal and high pulmonary artery pressure in terms of serum uric acid (*P* < 0.01).

### 3.3. Relation between severity of disease and serum uric acid and PAH

Table 5 shows relation between severity of disease and serum uric acid in studied patients.

**Table 5:** Relation between severity of disease and serum uric acid in studied patients.

Severity of disease	Mean level of uric acid	*p*-value
Low	3.52 ± 1.47	
Moderate	4.18 ± 1.57	0.012
High	6.10 ± 2.95	

**Fig. 1:**
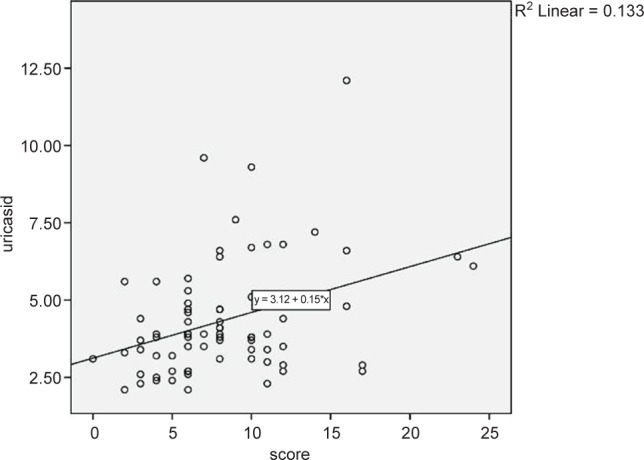
Coefficient correlation between serum uric acid and the severity of the disease.

Fig. 1 shows coefficient correlation between severity of disease and serum uric acid level in studied patients.

As shown in Table 5 and Fig. 1, significant correlation was seen between severity of disease and serum uric acid level in studied patients (*P* < 0.05).

Table 6 shows relation between severity of disease and pulmonary artery systolic pressure.

**Table 6:** Relation between severity of disease and pulmonary artery systolic pressure.

Severity of disease	Mean pulmonary artery systolic pressure(PASP)	*p*-value	Comparision between groups		*p*-value
Low	21.25 ± 2.50			Moderate	0.03
			Low		
Moderate	26.54 ± 9.94			High	0.02
		0.01			
High	31.25 ± 7.9		Moderate	High	0.001
				Low	0.03

As shown in Table 6, significant relation was observed between severity of disease and pulmonary artery systolic pressure *(p* = 0.01).

### 3.4. Relation between serum uric acid with duration of disease

**Fig. 2:**
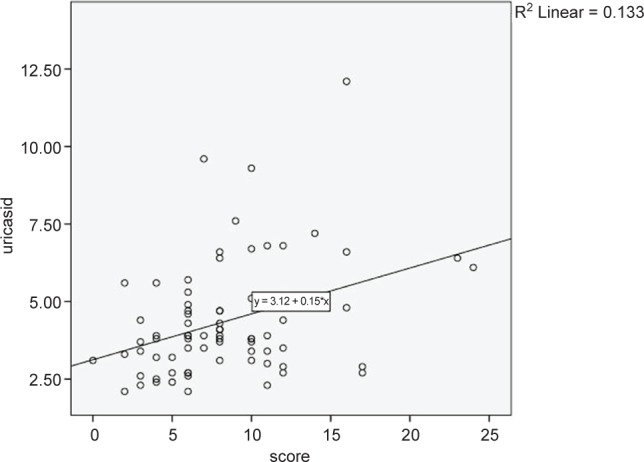
Coefficient correlation between serum uric acid level and the duration of the disease.

Coefficient correlation between serum uric acid level and duration of the disease showed that there was no significant correlation between serum uric acid level and duration of the disease. (p = 0.90, r = 0.016) (Fig. 2)

### 3.5. Classification of patients with normal and high PAH in terms of serum uric acid

Table 7 shows classification of patients with normal and high pulmonary artery systolic pressure in terms of serum uric acid level.

**Table 7:** Classification of patients with normal and high pulmonary artery systolic pressure in term of uric acid.

Serum uric acid level	Pulmonary artery systolic pressure (PASP)		Total
	Abnormal	Normal	
Normal	3(50%)	7(4.6%)	10(13.3%)
High	3(50%	62(95.4%)	65(86.7%
Total	6(100%)	69(100%)	75(100%)

It is clear from Table 7, among patients with normal and high pulmonary artery systolic pressure, 65 had high serum uric acid.

In addition, specificity, sensitivity, positive and negative predictive value of uric acid is 50%, 89.85%, 30% and 95.38%, respectively.

## 4. Discussion

Systemic lupus erythematosus is an autoimmune disease in which organs and tissues [12–28] are affected by the mediation of immune complexes and autoantibodies.

Contradictory results regarding the role of uric acid in high pulmonary arterial pressure and lack of sufficient studies especially in Iran and our region led to present study with aim of comparison of serum uric acid in patients with systemic lupus erythematosus with high and normal pulmonary arterial pressure. In this study, 75 patients with systemic lupus erythematosus were evaluated. All patients underwent echocardiography and serum level of uric acid was measured. Out of 75 patients, 10 (13.3%) had high serum uric acid (>7 mg/dl). Sheikh *et al.,* found that Hyperuricemia was detected in 16.1% of SLE patients. They reported that high incidence of hyperuricemia in SLE patients may be due to variant endogenous and exogenous mechanisms including inflammation, hypertension, and renal involvement which are prevalent in SLE patients. On the other hand, increased uric acid can worsen inflammation, hypertension and kidney disease, leading a vicious cycle [7]. Increased activity of enzymes such as xanthine oxidase in patients with SLE is another possible reason for higher prevalence of hyperuricemia in patients with SLE [7].

Moreover in our study, 8% of patients had high pulmonary arterial pressure. Kim *et al.,* [10] selected 114 patients with systemic lupus erythematosus. Pulmonary artery pressure was measured in these patients. The results showed 7.9% of patients had high pulmonary arterial pressure. Luo *et al.,* reported that the PAH occurrence was 4.9% in SLE patients [29]. In another study in Spain, among 245 patients with lupus, 12 cases (5%) had high pulmonary arterial pressure. Moreover, sensitivity, specificity, positive and negative predictive value of PAH was 100%, 97%, 70% and 100%, respectively.

The mean serum uric acid level in patients with and without pulmonary arterial hypertension was 2.13 ± 6.20 and 1.74 ± 4.19 *mg/dl,* respectively. There was a significant difference between patients with and without pulmonary artery disease in terms of mean serum uric acid level. Kim *et al.,* found significant association between serum uric acid and high pulmonary arterial pressure [30]. These results are consistent with our study which shows high increase of uric acid level in patients with pulmonary arterial pressure. Castillo-Grayson *et al.,* found a significantly increased risk of hypertension in subjects with hyperuricemia [31]. Zhang *et al.,* also reported serum uric acid was increased in more than one half of patients with pulmonary artery hypertension. Moreover, they reported that serum uric acid level is associated with severity of pulmonary artery hypertension and ventricular dysfunction [32]. Sheikh *et al.,* in another study found that high level of uric acid was significantly associated with hypertension independent of age and BMI. They believed that hyperuricemia has a main role in hypertension *via* several mechanisms. Uric acid activates the renin-angiotensin system and down regulates production of nitric oxide (NO), leading to vasoconstriction [7]. Moreover, uric acid stimulates production of pro-inflammatory cytokines which has a main role in the vascular remodeling that occurs in PH [9].

In our study, comparison between serum uric acid and echocardiography showed that the serum uric acid compared to echocardiography had a sensitivity 50% and specificity 89.85%. It seems that serum uric acid can be helpful in diagnosis of patients with lupus erythematosus. Therefore, there is no need for echocardiography in all patients. Kim also obtained same result and reported that the measurement of uric acid instead of echocardiography can be used to screen patients with high pulmonary arterial pressure [2, 10]. In another study, 114 patients with lupus were enrolled and echocardiography was performed. Pulmonary artery pressure was measured for all patients. The results showed that the measurement of uric acid (sensitivity 66.7% and specificity 96.2%) may be as an alternative to echocardiography for screening lupus patients with high pulmonary arterial pressure [7]. Xia *et al.,* showed that early identification and standard treatment of pulmonary arterial hypertension and SLE can improve prognosis of disease [33]. Min *et al.,* also confirmed previous studies and reported pulmonary artery pressure is an independent factor to predict survival in SLE patients [34]. Martínez [35] in 2011 evaluated 44 patients with systemic lupus erythematosus and measured pulmonary arterial pressure and uric acid in these patients. They observed that 10 cases had high pulmonary arterial hypertension and 34 cases normal pulmonary arterial pressure. They found that serum uric acid level can be predictor of pulmonary arterial pressure in patients with systemic lupus erythematosus. They also reported that high level of uric acid can be as predictor of high pulmonary arterial pressure in the future [31]. But Castillo-Martínez in 2016 conducted a study on 156 systemic lupus erythematosus (SLE) patients and no association was found between pulmonary hypertension and markers such as serum uric acid. This study indicated that serum uric acid at one point in time can’t use to predict pulmonary hypertension [31].

Uric acid as the final product of oxidation of purine is associated with oxidative metabolism disorder. Moreover, it may be associated with severity of disease and prognosis of patients with pulmonary arterial pressure and different etiology [36-38]. In our study, there is a significant correlation between serum uric acid level and severity of disease. So that with increasing severity of the disease, serum level of uric acid has also increased. This means that the measurement of serum uric acid is as a prognostic factor in all patients with lupus. These results are consistent with the findings of the study by Castillo-Martínez D. Moreover, Kim *et al*., showed that serum uric acid level is correlated with functional severity. Since high level of serum uric acid predicts poor outcomes, it may carry prognostic value, although it is considered strictly a biomarker [9].

Moreover, in our study, the increase of pulmonary arterial pressure was significantly associated with the severity of the lupus disease which was not consistent with Castillo *et al*., study in 2011 [35]. However, our results were consistent with Min HK study in Korea [34]. Prabu *et al.,* found an association between pulmonary arterial hypertension (PAH) with rapid deterioration and poor prognosis of SLE [38]. Luo *et al.*, reported that patients with higher scores of SLE disease were favorable to PAH [13]. It seems that pulmonary hypertension as an unusual manifestation of lupus with high morbidity is life threatening. Moreover, it is more common in patients with severe disease [39, 40]. However in our study, the serum level of uric acid does not correlate with duration of lupus disease which is consistent with Kim *et al.,* study [10]. Kamel *et al.,* obtained similar result and reported that no significant correlations were observed between pulmonary artery pressure and duration of disease [41].

## 5. Conclusion

According to results of this study, a significantly increased level of serum uric acid was observed in patients with pulmonary arterial pressure. Hence, serum uric acid level could be a prognostic marker of pulmonary arterial pressure in SLE patients which correlates with disease severity. It also would help to reduce clinical demands for echocardiography in patients with normal uric acid levels.

## Conflicts of interest

The authors declare no conflict of interest.
